# Qualitative assessment of user experiences of a novel smart phone application designed to support flexible intensive insulin therapy in type 1 diabetes

**DOI:** 10.1186/s12911-016-0356-6

**Published:** 2016-09-15

**Authors:** Brigid A. Knight, H. David McIntyre, Ingrid J. Hickman, Marina Noud

**Affiliations:** 1 Queensland Diabetes and Endocrine Centre, Mater Health Services, Brisbane, Australia; 2 Lady Cilento Children’s Hospital, Brisbane, Australia; 3 School of Medicine, University of Queensland, Brisbane, Australia; 4 Mothers and Babies Theme, Mater Research Institute – University of Queensland, Brisbane, Australia; 5 Mater Research Institute, University of Queensland, Brisbane, Australia; 6 Department of Nutrition & Dietetics, Princess Alexandra Hospital, Brisbane, Australia

**Keywords:** Intensive management, Diabetes technology, iphone, Applications, Self-management

## Abstract

**Background:**

Modern flexible multiple daily injection (MDI) therapy requires people with diabetes to manage complex mathematical calculations to determine insulin doses on a day to day basis. Automated bolus calculators assist with these calculations, add additional functionality to protect against hypoglycaemia and enhance the record keeping process, however uptake and use depends on the devices meeting the needs of the user. We aimed to obtain user feedback on the usability of a mobile phone bolus calculator application in adults with T1DM to inform future development of mobile phone diabetes support applications.

**Methods:**

Adults with T1DM who had previously received education in flexible MDI therapy were invited to participate. Eligible respondents attended app education and one month later participated in a focus group to provide feedback on the features of the app in relation to usability for patient-based flexible MDI and future app development.

**Results:**

Seven adults participated in the app training and follow up interview. App features that support dose adjustment to reduce hypoglycaemia risk and features that enable greater efficiency in dose calculation, record keeping and report generation were highly valued.

**Conclusions:**

Adults who are self managing flexible MDI found the Rapidcalc mobile phone app to be a useful self-management tool and additional features to further improve usability, such as connectivity with BG meter and food databases, shortcut options to economise data entry and web based storage of data, were identified. Further work is needed to ascertain specific features and benefit for those with lower health literacy.

## Background

Self management is the cornerstone of type 1 diabetes (T1DM) management and patient-based flexible multiple daily injection (MDI) therapy requires the individual to perform a number of (often complex) mathematical calculations [1]. To calculate the prandial insulin dose, the carbohydrate count must first be calculated for a range of foods, requiring individuals to interpret carbohydrate data from food labels, nutrition databases and other published sources. Once the carbohydrate quantity is determined, the insulin:carbohydrate ratio is then applied, either by division or multiplication (depending on the method of carbohydrate counting). Subsequently, a dose correction is determined by subtracting target blood glucose (BG) from the prandial BG and dividing by the correction factor. Numeracy skills are essential for effective flexible MDI therapy and lower numeracy scores are associated with lower self-efficacy and higher glycosylated haemoglobin (HbA1c) [2, 3]. Sussman and colleagues compared manual insulin dose calculations to an automated bolus calculator (ABC), and found 63 % of dose determinations conducted manually were incorrect compared to 6 % of calculations via an ABC [4].

Automated bolus calculators (ABCs) integrated with BG meters and insulin pumps have enabled safe and effective bolus determination as demonstrated by improvements in 1) glycaemic control [5–7], 2) treatment satisfaction [7, 8], and 3) confidence in dose determination [4, 9] and reduction in 1) frequency [10] and fear of hypoglycaemia [9], 2) dose calculation errors [4] and 3) treatment burden associated with mathematical calculations and record keeping [11].

In addition to managing the prandial dose calculation, effective ongoing management of patient-based flexible MDI therapy requires the person with diabetes to undertake additional self management tasks which add to the daily burden of diabetes management. These tasks include counting carbohydrates (which involves measuring foods, interpreting food labels, researching unfamiliar foods, performing an estimate of carbohydrate count when eating out), monitoring blood glucose levels (which involves washing hands prior to testing, taking care with meter and test strip handling, remembering to monitor prior to eating, pre bed, prior to driving and when feeling symptomatic with hypoglycaemia), learn the impact of physical activity and alcohol on blood glucose and make anticipatory adjustments, record BG and additional pertinent diabetes management information (such as carbohydrate, alcohol, activity, stress, illness) in a diabetes diary, and effectively collaborate with health professionals.

The BG or diabetes diary, which is often paper-based, is an essential component of flexible MDI, as it enables comparison to BG targets and enables BG trends to be analysed to inform proactive changes in insulin formula. Despite the importance of the BG diary, compliance with BG diary recording is often poor [12]. Use of an electronic hand held diary has been shown to improve compliance with record keeping, compared to the ‘traditional’ paper-based diary [13] and the combining of dose calculator and BG diary recording functionality may explain the satisfaction and preference for ABCs [4]. Some ABCs enable users to record additional pertinent data (such as exercise and illness), usually in pre-determined text format. Free text data recording is not available in ABCs.

Integration of bolus calculator functionality into mobile phones has emerged, in some cases, with SMS messaging and telemedicine [14]. In addition to the dose calculator and BG diary functionality of ABCs, mobile phones have added advantage of ‘connectivity’, enabling more efficient communication of data with health professionals providing self management support. Improved motivation, self management reflection and communication between patient and health professional has been observed with use of mobile phone diary apps [15], highlighting the benefits of this technology in self management support.

In Australia, there are over 30 million mobile phones registered [16], which is more than one per person and more thank half (68 %) use mobile phone apps [17]. The ubiquitous nature of mobile phones ensures universal access to this technology, potentially enabling a greater number of individuals to access an ABC with BG diary features.

The RapidCalc mobile phone app was developed locally, as an adjunctive tool to support a specific subset of adults with T1DM – those who have been trained in flexible insulin self management using carbohydrate counting and insulin:carbohydrate ratios. The app has been developed specifically to provide a phone-based platform to support the following self-management practices: bolus dose determination (based on individual insulin adjustment algorithms), diabetes diary recording, report generation and communication with health professionals. The app bolus calculation equation is consistent with ABCs, with the calculation for bolus doses determined from three calculations: 1) bolus for carbohydrate, based on insulin:carbohydrate ratio, 2) correction bolus based on prandial glucose, insulin sensitivity and target BG and 3) insulin on board (IOB) based on residual insulin from previous bolus dose(s). Rapidcalc also incorporates the following features, which differentiates it from ABCs: 1) free text option for recording pertinent details relating to a specific events, 2) food photograph option, that enables the user to save a food photograph, time stamped with the relevant carbohydrate count in the diabetes diary, 3) variable options for bolus adjustment for exercise, 4) ‘Reverse calculator’ which calculates the carbohydrate required when BG is below target, 5) omission of correction bolus for high BG due to an antecedent hypoglycaemic event, or when consuming a significant quantity of alcohol and 6) data export capabilities via email.

To our knowledge, there are currently no other phone-based bolus calculator applications which incorporate these features.

The aim of this study was to obtain user feedback on the usability of the RapidCalc app in adults with T1DM already experienced in flexible MDI, with a view to informing further development of this application and identifying user preferences with this emerging technology. We sampled from graduates who had recently completed structured education in flexible insulin self management education to explore the specific features that this specific type of insulin user would want from a phone based app to support day to day diabetes management.

## Methods

### Participants

The interview group consisted of 7 participants who had previously received flexible MDI education program by attending the Dose Adjustment for Normal Eating (DAFNE) program. The DAFNE program aims to equip graduates with evidence based insulin self-management skills based on carbohydrate counting and insulin algorithms, and includes insulin adjustment strategies for physical activity, illness and when consuming alcohol, and pattern evaluation of BG trends to inform ongoing insulin adjustment to achieve target glucose levels. Each DAFNE course is delivered in a group setting, over 5 consecutive days. DAFNE graduates were chosen, firstly, as they have received the same insulin self-management education and secondly, because they have had considerable experience contributing their opinion alongside others with T1DM in a group setting. Graduates who had completed DAFNE education in the most recent 13 months were invited by email to participate. The inclusion criteria were: age 18–65 years, access to an iphone or iPod touch, able to attend a 1-2 h training session at study commencement and 1–2 h group interview at the end of the trial period, willingness to email RapidCalc results to the study centre once per week for the first four weeks of the study, using insulin Glargine (Lantus), Insulin Detemir (Levemir) or NPH (Humulin NPH, protaphane) insulin for basal insulin and Insulin Glulisine (Apidra), Insulin Lispro (Humalog) or Insulin Aspart (Novorapid) for bolus insulin, able to inject insulin for all meals and large snacks, able to test BG at least 4 - 6 times/day, HbA1c 53 - 86 mmol/mol (7–10 %), absence of end stage diabetes complications or other serious medical condition except for well managed coeliac disease, thyroid disease or asthma (not requiring oral steroids) and able to read and speak English. Criteria for exclusion were: hypoglycaemia unawareness, major psychiatric illness that prevents interaction in a group setting, pregnancy or breast feeding and use of an insulin pump.

### Procedures

Four weeks prior to the app education session, participants were instructed by phone or email how to load the app on their phone. Participants were asked to maintain a paper-based BG diary for 4 weeks, with weekly transmission to the study coordinator. The purpose of this was to support participants in optimising insulin algorithms prior to app use.

### App education session

Education on the use of the app was conducted by the app developer in a group setting with all participants present. A diabetes educator (BK) was in attendance to support programming of the app with each user’s existing insulin dose algorithms. Participants started using the app following this session. Participants were invited to contact the diabetes educator (BK) for assistance with dose adjustment at any time until the focus group meeting. One month after app education session, all participants were invited to return for the focus group.

### The focus group

The focus group was led by a diabetes educator (MN) who was not involved in development of the app or in the app education session. The open ended interaction of a group interview was chosen to stimulate thought and emotions, to reveal material which may not ordinarily be expressed in an individual interview. A semi-structured interview format was used, focusing on the attributes of usability, which include learnability, efficiency, memorability, errors and satisfaction [18]. Interview prompts were devised within the research team, which was comprised of experienced clinicians and researchers and focus group questions are summarised in Appendix A. The aim was to develop themes to address the aims of the study without being deemed leading or judgemental.

At this visit, app data exports (data reports which show data entered by each user) were obtained for each participant to identify the duration of app use. The interview was audio recorded, then transcribed verbatim. Thematic analysis of the focus group followed the structured approach outlined by Braun & Clarke [19] where themes were identified according to the group discussion and not limited to the usability attributes. Themes were coded according to specific app features to highlight the features that participants found useful or not. The transcript was reviewed by two reviewers (BK & MN) independently, who met to address discrepancies and agree on themes.

## Results

Fifteen DAFNE graduates were sent an invitation to participate and nine graduates expressed interest, however 2 were unable to participate due to scheduling conflicts. Seven adults (5 female) with mean (SD) age of 36 (7) years and mean duration of diabetes 27 (7) years, attended app education and a 2 h focus group. Participant characteristics are summarised in Table 1. Six of the seven participants used the app for the duration of the study period. The one participant who had not used the app for the duration of the study indicated that she had failed to sustain regular BG monitoring during the study, therefore ceased using the app.

**Table 1:** Participant characteristics

Participant number	Age (years)	Gender	Diabetes duration (years)
1	30–34	F	20
2	30–34	M	26
3	30–34	F	21
4	30–34	M	25
5	30–34	F	27
6	35–39	F	34
7	50–54	F	38

Three main themes were identified from the focus group: 1) bolus calculator features and trust, 2) diabetes diary and report features and 3) satisfaction and control. Within each theme, sub-themes are described, with respondent quotes coded according to gender, number identifier, age category and diabetes duration: for example, F5 (A40-44, D20) refers to female number 5, aged 40–44 years, with 20 years diabetes duration.

### Bolus calculator features and trust

Users programmed the app with their personal insulin algorithms with the help of the study team and none of the group reported difficulty in the set up process. Six participants reported that they trusted the accuracy of the dose calculation via the app, though five reported they like to ‘check the dose’ recommended by the app, against manual calculations. One participant (F1, A35-39, D27) rarely checked the dose, acknowledging that "I'm one of those people in the population that would go, 'I don't have to think, just tell me what to have.' “ This person went on to add that she trusted the app more than her own calculations, claiming that “ the app is a lot more consistent that I am” and “It's better than guessing in the complete dark - it's less dark." She went on to acknowledge that her trust in the app meant that she didn’t check her ratios (confirm suitability of current dosing algorithms), which she admitted did not enable ‘fine tuning’ and ongoing dose adjustment. F5 (A30-34, D25) described how the app made it easier for her to remember the insulin:carbohydrate ratio to use for specific meals, especially after a change in prescription for insulin:carbohydrate ratio.

Four participants described over-riding the dose, not because of a lack of trust, but for specific reasons, such as when physically active or when snacking. There was group agreement with F5 (A30-34, D 25), who reported "It's not that I don't trust it, it's just that I need to over-ride it." M1 (A30-34, D26) highlighted the problem with the preset time frames for dose algorithms in the app: "My ratios didn't fall into time frames provided by the app. I usually use the 1:1 ratio at lunch and a 2:1 ratio in the evening, so......if I had a snack in the afternoon, I usually use the 1:1.... but if I have the snack between 4 and 5 (pm) and that falls within the dinner… so I would be ignoring the dose that it was calculating because I thought no, that is twice as much as I want to be having." The 4 participants who had to override the app due to preset time frames for the insulin algorithms stated a preference for customisable time settings for insulin algorithms.

Three participants found the IOB feature useful in reducing the risk of hypoglycaemia. F1 (A35-39, D27) described this feature as "The best thing in the world." Though considered useful, many found that the IOB was misleading when meals and snacks were eaten in close proximity. The IOB is calculated based on total residual bolus insulin, therefore may overestimate the IOB in instances where meals/snacks are eaten in close proximity. Five participants agreed that they needed to over-ride the bolus suggestion when meals were consumed in close proximity.

The reverse calculator, which determines the carbohydrate to be consumed for BG below target, was used by 4 participants. Two users described specific instances where this feature protected against hypoglycaemia, or prevented over-treatment of hypoglycaemia. F3 (A30-34, D21) states, "What I love… was that it told me how much to have and I didn't overeat. The number of times that I overeat from hypos is ridiculous; it would be 99 % of times.” Four participants reported that the carbohydrate supplementation suggestion from the reverse calculator tended to be lower than their usual guidelines for hypoglycaemia management (15 g rapid acting carbohydrate). M2 (A30-34, D26) was particularly concerned when the app suggested dosing insulin for some of the hypo treatment: "Cause I found when I would be 3.4 or 3.3 (mmol/l), the DAFNE rules would say 1.5 CPs (carbohydrate portions) and I'd put in 1.5 CPs and it would be telling me to take one unit of insulin, which I obviously wasn't going to do.”

The exercise bolus adjustment feature, which can be programmed to reduce the bolus dose in the setting of physical activity, had been used by 4 participants. All agreed that adjusting insulin to manage BGLs around exercise was challenging. F4 (A30-34, D20) sums up her experience with the exercise feature, “I don't know how I'm reacting to exercise because I don't pay enough attention to it” and F2 (A35-39, D34), "I always muck it up somehow. I never seem to crack it.” There was general agreement that exercise planning was difficult and that in order for “any app to work,” each user had to first understand their own exercise effect and develop management strategies with carbohydrate supplementation and/or insulin reduction. Two of the group reported that they used the exercise feature regularly: that it was beneficial and that the feature served as a prompt to think about changing bolus doses when they were more physically active than usual. Three participants agreed that a basal dose adjustment prompt for prolonged exercise would also be helpful in instances where exercise is of long duration.

The alcohol bolus adjustment feature, which withholds the correction bolus after alcohol consumption, was considered by the group as an important feature, however there was disparity in the quantity of alcohol that represents the threshold for when this feature should be chosen. All users agreed that being able to record alcohol consumption (even for small quantities, when no dose change was programmed) was important, to enable reflection on the effect of alcohol on BG levels.

Blood glucose data is entered manually into the app to determine the bolus dose. The entire group agreed that it would be beneficial to have a BG meter that could transmit glucose data directly to the app, eliminating the need to manually enter glucose data into the phone. Responses to this option were “That would be brilliant”; “I would do a dance about that one" and "That would be awesome" emphasize the overwhelming desire for this feature.

### Diary report features

There was an overwhelming group consensus that the app was a ‘great’ record keeping device, due to 1) the convenience of entering data onto a smart phone and 2) the fact that participants were rarely without their phone, enabling “better record keeping” compared to previous diary methods and other apps that participants had used.

Participants expressed the desire to retrospectively edit data pertaining to their diabetes management or food consumed. M1 (A30-34, D26) described, "It drives me mad how you can't put an entry in after (an event) at any time of the day. Then I don't bother putting it in. You just wouldn't do it: (you) just forget about it." There was also a consensus that the ability to record a ‘true’ account of diabetes management history (using free text options) and record pertinent events was a valuable feature. Participants raised the idea of a ‘shortcut’ button to enable easy recording of a pre-determined list of events, in addition to the current free text option, to record what they considered to be pertinent and useful information, when later reviewing BG trends. To further individualise the app, many felt it would be useful to be able to personalise screen displays by allowing removal (or cloaking) of features to simplify the user interface.

Three participants found the Rapidcalc method for creating diabetes diary reports, using a macro to convert raw data into a diabetes report was difficult, and that not being able to easily generate this BG diary data for their treating clinician was a problem. The group agreed that web based storage of settings and BG diary data would be a more effective way to manage their diabetes diary, both for their own reflection and when consulting their health care provider. Some participants referred to web based storage of other phone data and that web based storage of the RapidCalc settings on the phone would also be useful to ensure data safety in the event of loss of device.

There was group agreement on the desire for food database connectivity, to assist with carbohydrate counting and enable foods consumed to be easily recorded in the diabetes diary. Some participants suggested that it would be helpful to ‘register’ favourite foods in the set up process, enabling favourite foods to be recorded in the diary via a shortcut menu. Participants also felt that it was important to capture specific information about food when reviewing BG trends and some had used the photograph feature for this purpose.

### Satisfaction and control

There was group consensus that the app made bolus calculation easier and quicker, with one participant (F1, A35-39, D27) reporting that "the application worked for me in that there was less thinking involved" and that BG control improved from the start of app use. The group agreed that the app provided an improved means of diabetes record keeping compared to previous methods. Two participants also reported improved BG control since using the app.

Three participants, reflected on improved satisfaction, compared to their experience with other apps. F1 (A35-39, D27) reported, “I was using another diabetic app and it wasn't maintainable for me” and F5 (A30-34, D25), “I find (the app) so user friendly compared to other apps.” M1 (A30-34, D26) reported, "I quite enjoyed using it," and F1 (A35-39, D27), "It has offered me something that nothing else in my busy-ness and my avoidance has offered me. " The entire group found the app to be a useful adjunct to their diabetes management and a number reported they enjoyed using it.

## Discussion

Users of the Rapidcalc app developed a trust in the app calculation rapidly and described improved efficiency and efficacy of day to day insulin management compared to other methods of dose calculation and recording, and recommendations to further improve usability and reduce the burden of management were identified.

The attributes of usability for which the group were able to give feedback were errors, efficiency and satisfaction. Errors were not detected and trust in the app dose calculation was determined early due to ‘dose checking’ against manual calculations, which the users were accustomed to doing prior to app use. Early establishment of trust in the dose calculation may be due in part also to the way that the components of the bolus dose are displayed on the screen, enabling quick verification of bolus dose components and total dose. This is in contrast to Shepard and colleagues [20], who found that pump users did not trust an ABC (the Personalised Glucose Advisory System) when they could not determine how the dose was calculated. The issue of over-riding the app bolus calculator dose highlights the knowledge and experience in this cohort to understand other factors that impact on BG levels and reflects the active role these users take in the insulin dose adjustment process. Users who lack the knowledge and experience to confidently verify bolus insulin doses may not develop trust as readily.

Users commented on the quick and easy access to dose calculation and diary recording on a device that most people carry on their person, suggesting greater efficiency compared to alternative devices, and suggestions were offered to improve efficiency further. Features that could ‘save time’ and reduce hypoglycaemia risk were valued and the specific app features identified as useful were IOB, reverse calculator, exercise and alcohol bolus modifiers and phone-based BG diary. Though this was not measured, improved glycaemic control was also reported by some participants.

Users were able to give feedback on learnability in the setting of optimal access to health professional support. With appropriate training, users found the app easy to learn and use. First time users who do not have health professional support may struggle with learnability, especially if they are not familiar with flexible MDI strategies, such as carbohydrate counting and use of insulin dose algorithms, as the app requires the user to input specific insulin algorithms based on carbohydrate counting. Given that this app advises insulin dosing, it is expected that users would collaborate with their health professional in the programming and familiarisation process. Participants felt that they could not comment on memorability as they had not experienced resuming app use after a period of discontinuance.

Much of the discussion focused on app features that the group found useful and how the app could be further improved. The IOB feature is a novel concept for those who had not used an ABC previously and there was a positive response about the benefits of this feature in reducing hypoglycaemia risk. The reverse calculator was seen to be beneficial in controlling the tendency to ‘over treat’ hypoglycaemia. Over treatment of hypoglycaemia is a common practice and impacts negatively on HbA1c [21]. The discrepancy between ‘usual’ hypoglycaemia management protocol and the calculation from the app is not surprising, given that the standard treatment for hypoglycaemia is based on a low glucose threshold (≤ 3.5 mmol/l) and fixed quantity of carbohydrate (15 g), whereas the app mathematically calculates the relative carbohydrate quantity required to return the BG to target range. This issue is likely to be a concern with other users as well and highlights the importance of hypoglycaemia management education.

The bolus adjustment feature for exercise and alcohol were found to be helpful in reducing hypoglycaemia risk in those who used these features, though users felt that the threshold for bolus reduction in the setting of alcohol consumption needs to be individualised. Regarding bolus modifications for exercise, there was an overwhelming consensus that BG management around exercise was difficult and that individual requirements for exercise needed to be established before being confident to use the app to modify insulin doses for exercise. Users found that the exercise and alcohol bolus adjustment features served as useful prompts to ‘consider’ dose adjustment in the these settings. Fully adjustable time frames were also preferred, to enable dose algorithms to be tailored to individual requirements for meals and snacks. Pre-set time frames for insulin algorithm settings appeared to increase the burden of dose determination in individuals who use varied algorithms through the day.

The idea of connectivity between the app and food composition databases, to enable carbohydrate data to be sourced and recorded, was favoured by the group: a preference that has been demonstrated in other reports [15, 20]. Connectivity with the internet was also favoured by the group, as a means to enable the diabetes diary data to be ‘securely’ stored and potentially able to be accessed remotely by health professionals.

Users placed high value on the diabetes diary and felt that it should reflect a full and true account (including retrospective data edit capability) of diabetes management, confirming that users consider a comprehensive diary to be a valuable tool in their diabetes management. This desire to retrospectively edit diabetes diary data has also been reported by Arsand and colleagues [22]. Users felt it was important to record all data they perceive to be pertinent, either by free text or with ‘personalised’ buttons to ‘economise’ data entry. Users of the Personalised Glucose Advisory system [20] also reported that the bolus calculator should take into account and record a broad range of factors affecting BG levels, such as physical activity duration and intensity, consumption of higher protein and higher fat meals, hormonal changes relating to the menstrual cycle, breastfeeding, stress, shift work and alcohol consumption. The concerns about data safety and having easy access to diary data lends further support to the importance that users place on diabetes diary data and the sharing of this information with health care professionals.

Lawton & colleagues [11] voiced the concern that some users of ABCs will fail to verify bolus doses or reflect on glycaemic outcomes to inform ongoing insulin dose titration. This is a valid concern. ‘Dose checking’ did drop off in this cohort, as seen in the Lawton cohort, however one would expect this attrition, given that users have confirmed that the calculations can be trusted. In this cohort as well as Lawton’s, individuals were identified who exhibited either a ‘blind trust’ in the bolus calculator, or did not know how to adjust the insulin algorithms, which is a concern if the algorithms are not optimised. Lawton’s concern that dependency on the bolus calculator may lead to deskilling, abandoning diabetes diary reflection and proactive titration of insulin doses and reliance on the bolus calculator, leaving users potentially unable to determine doses in the event of meter failure is also valid and highlights the importance of ongoing education and support, before and after the introduction of ABC’s.

A recurring theme across a number of features was the desire for functionality that reduces the burden of day to day management: in diabetes diary recording and carbohydrate counting, with shortcut buttons and BG meter and food database connectivity, personalisation of user screens and easier access to BG diary data. This highlights that role that ABCs can play to reduce the burden of day to day management, however care should be taken to ensure that ABCs are not offered in place of structured education in insulin self-management, a process that is difficult to ‘short cut’. Others have stressed that when ABCs are used, comprehensive education in flexible MDI should remain the key focus of self – management [11, 23], with education and support on ABC use delivered by health professionals familiar with ABC functionality. Structured education and ongoing support for flexible MDI using an ABC should include, at the very least, the following: 1) appropriate education on patient-based flexible MDI, including carbohydrate counting, 2) comprehensive education on programming and use of the dose calculator, delivered by health professionals experienced with these devices, 3) pattern evaluation education using the bolus calculator diary and graphs and 4) regular and ongoing insulin optimisation support from health professionals experienced in use of ABCs and a meter failure plan.

### Limitations

The main limitation of this study is the small number of participants and one focus group. We deliberately targeted adults with T1DM who were managing their diabetes in a specific way, using advanced skills that require a certain degree of health literacy and self-directed care. Using DAFNE graduates ensured that all participants had received similar flexible MDI education, enabling us to focus on the patient experience with the app in those who have received flexible MDI self management education. All DAFNE graduates have experienced nearly 40 h of group interaction and hence we were confident of the ability of focus group participants to not be ‘led’ by others in the group, or for the group to be dominated by any individual. Despite the small number of participants, we feel feedback from this focus group has yielded valuable user experiences on specific app features in individuals self-managing flexible MDI. We are not able to comment on the usability of this app other patient groups, where flexible MDI education has not been provided, or in those with low health literacy.

The recruitment of DAFNE graduates yielded a cohort that is more confident with carbohydrate counting and patient-based insulin adjustment and the trust in the app calculation and willingness to over-ride the bolus suggestion for special circumstances observed in this cohort is most likely enhanced by a familiarity with insulin adjustment terminology and patient-based insulin dose adjustment strategies. The process of confirming the dose calculation and over-riding a dose based on prior experience may not apply to users who are not confident with insulin dose adjustment strategies and this may have an eroding effect on confidence and willingness to use the app in instances where adequate education and support are not available.

The focus group participants all had long standing diabetes (average 27 years, range 20–38 years), however, this selection bias is not expected to impact on how the app may be perceived or used. Those newly diagnosed with T1DM are taught similar concepts in patient-based insulin optimisation from diagnosis, hence are also expected to be able to successfully use the app, providing insulin algorithms are adjusted as insulin requirements change during the honeymoon phase.

The short duration of follow up limits the ability to comment about retention or efficacy of this device when it is no longer novel or resumption of app use after a period of discontinuance. Given that the app performs the dual function of dose calculation and diabetes diary recording and has the potential to reduce the burden of diabetes management, we feel that users who are motivated to self-manage patient-based flexible MDI and maintain a diabetes diary will continue to use this app in preference to paper-based approaches and other mobile phone apps currently available. For those resuming app use after a period of discontinuance, the ‘help’ screens within the app may assist with memorability. The inclusion of pattern recognition software has been suggested [11] and this may reduce attrition associated suboptimal control due to inappropriate insulin algorithms.

Flexible MDI therapy is also taught outside of the DAFNE program [24–26], and though there are differences between programs in terms of mode of delivery and course duration, these programs all address what are considered to be the ‘core’ elements of insulin self-management education, namely, carbohydrate counting, insulin dose algorithms, adjustment for illness and exercise and proactive dose adjustment of insulin algorithms, and programs generally require users to manage dose calculations manually [27]. We feel that the insights from this focus group can be extrapolated to other patients who have received flexible MDI education outside of the DAFNE program.

Insulin users with low health literacy may gain as much, or greater benefit from using a phone app for dose calculation, to reduce risks associated with dose calculation errors and improve communication of data with health professionals. As with this study cohort, care would need to be taken to ensure that the appropriate carbohydrate counting education is provided, however modifications to the app, such as pattern recognition and prompts to contact health professionals are potential exciting directions for future research with a broader user group.

## Conclusions

Overall, users were satisfied and enjoyed using the app to manage their diabetes and trust in the bolus calculator was developed relatively quickly. The ability to maintain a true and complete diabetes diary that enables users to record a range of factors in daily management is important to adults with T1DM who are actively engaged in their insulin self-management. Features such as personalised user screens, shortcut buttons for data entry, and food database and meter connectivity, were highly valued for their potential to reduce the burden of day to day management. Also favoured were options to increase accessibility to a full and accurate account of diabetes management in the diabetes diary that is easily accessible to users and their health professionals. The importance of comprehensive education, both in terms of patient-based insulin dose determination, as well as ongoing dose titration is not diminished or substituted by use of a bolus calculator. These devices should be considered as tools to reduce the burden of flexible MDI and should supplement sound flexible MDI education.

This focus group has provided valuable insights into the features of a smart phone-based app that adults with T1DM feel are important to support effective patient-based flexible MDI. Longer term follow up of patient experience and clinical efficacy of mobile phone apps incorporating these features warrants further investigation.

## Appendix A

Focus group questions

**Table:**

Questions	Attribute
Tell me about your experiences using the rapidcalc app Prompts if needed: • Challenges • Benefits of app	Satisfaction
Describe your experience with learning how to use the app (general question first, then more specifically,…) Prompts if needed: • Inputting settings • Use of help screens • Using dose calculator	Learnability
Did you feel you could trust the bolus calculator? Prompts if needed: • How closely does the application suggested dose compare with your usual bolus calculations? ▪ For meals ▪ For snacks	Errors
How does the app influence your efficiency in: • Dosage calculation? • BGL diary recording? • Exercise adjustments? • BGL diary reflection? • Communication of your diary with another person (eg. your health professional)	Efficiency
Specific features- what is your experience with specific app features: • IOB • Reverse calculator • exercise feature • history page • graphs	Efficiency satisfaction
If you were to have a period of not using the app, do you think that you can easily pick it up again and remember how to use it?	Memorability
What would you ask the developers to change? Given the opportunity to build a new application from scratch, what would you put in to make it better?	Future improvements
Do you plan to continue to use rapidcalc?	Satisfaction

## Abbreviations

ABC: Automated bolus calculator
BG: Blood glucose
CP: Carbohydrate portion
DAFNE: Dose adjustment for normal eating
HbA1c: glycosylated Haemoglobin
Hypo: Hypoglycaemia
IOB: Insulin on board
MDI: Multiple daily injection
T1DM: Type 1 diabetes mellitus
